# Supplementary material for: Quantum fluctuations lead to glassy electron dynamics in the good metal regime of electron doped KTaO3
Source: Nat Commun. 2024 May 7;15:3830. doi: 10.1038/s41467-024-47956-4 (PMC11076559; doi:10.1038/s41467-024-47956-4)
Supplement: Supplementary file 1 — Supplementary Information [file 41467_2024_47956_MOESM1_ESM.pdf]

Supplementary Information

List of contents

Note 1. Calculation of electron's mean free path.
Note 2. Sheet resistance vs temperature plot.
Note 3. Stretched exponential behavior of resistance relaxation in the light off stage.
Note 4. Power law behavior of persistence photo-resistance relaxation time (τ).
Note 5. Experiment with red light.
Note 6. Long-time relaxation of persistence photo-resistance.
Note 7. Deviation of persistence photo-resistance relaxation time (τ) from activated behavior at low temperature.
Note 8. Dielectric loss in pristine $KTaO_3$.
Note 9. Correspondence between the appearance of polar nano regions and photo-doping effect.
Note 10. Raman measurement on a metallic oxygen deficient KTO.
Note 11. Presence of potassium vacancy.
Note 12. Berry phase calculations.
Note 13. Toy model of complex inter-band electronic relaxation mediated by a glassy bath.

Supplementary Note 1: Calculation of electron's mean free path

The Fermi surface of the electron doped KTO [1] comprises of three ellipsoids of revolution centered at Γ point (see Supplementary Fig. 1a) with major axis $k_{F,max}$ and the minor axis $k_{F,min}$ transverse to it. In the presence of magnetic field along [001] crystallographic axis, electrons traverse around the extremal orbits in the momentum space as shown in the Supplementary Fig. 1b, leading to the observation of SdH oscillations in magnetoresistance. Please refer to our earlier work [2] for SdH oscillation data on the same sample which is being investigated in the current study. These oscillations are periodic in $1/B$ whose frequency F_{SdH} is related to the area of extremal orbits A_{ext} as given below [3]

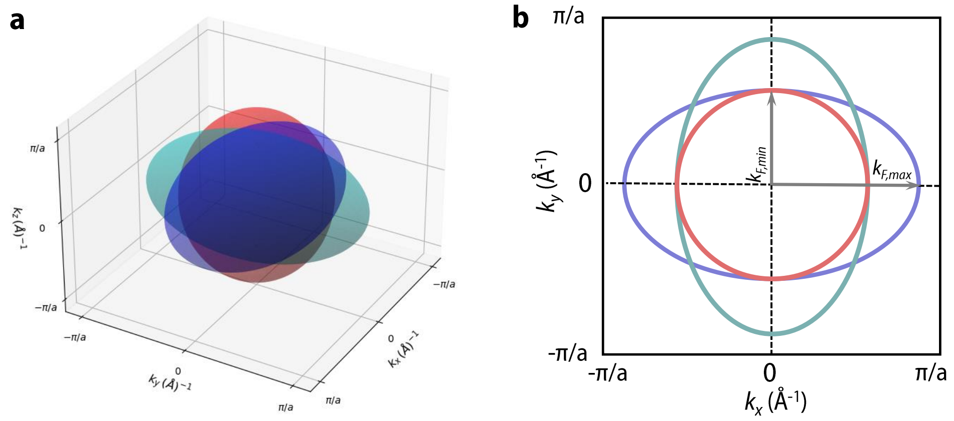

Supplementary Figure 1: **a.** Ellipsoidal Fermi surfaces of electron doped KTO. Here $k_{F,max}$ was taken to be 1.541 times $k_{F,min}$ for plotting. **b.** Cross section of the Fermi surface with the plane k_Z=0

$$F_{\text{SdH}} = \hbar A_{ext}/2\pi e \tag{1}$$

The extremal orbits corresponding to the two ellipsoids directed along k_x and k_y axis are ellipses with area equal to $\pi k_{F,min} k_{F,max}$, whereas the extremal orbit corresponding to the ellipsoid along the k_z direction is circular with an area $\pi k^2_{F,min}$ (see Supplementary Fig. 1b). Further, from the literature it is known that [4, 1]

$$k_{F,max}/k_{F,min} = 1.541 \tag{2}$$

for electron doped KTO, which would further imply that there are more electrons around elliptical orbit than those orbiting around circular orbit [5]. From above fact, we assume that the main frequency of SdH oscillation comes from electrons orbiting around the elliptical orbit and therefore A_{ext} in Supplementary Eq. (1) is taken to be the area of the ellipse $\pi k_{F,min} k_{F,max}$. From the SdH analysis, F_{SdH} for our oxygen deficient KTO is found to be 12.8 Tesla [2]. Putting this value in Supplementary Eq. (1) and using the Supplementary Eq. (2), we first calculate $k_{F,max}$ and $k_{F,min}$ individually and then 3D carrier density is determined. For this we note that the total volume occupied by three ellipsoid is $\text{V}_k = 3 \times (4\pi/3) k^2{}_{F,min} k_{F,max}$ from which the carrier concentration n can be calculated as [5]

$$n = (k^2{}_{F,min} k_{F,max})/\pi^2 \tag{3}$$

Putting the value of $k_{F,max}$ and $k_{F,min}$ in the above Supplementary Eq. (3), the 3D carrier density n for the oxygen deficient KTO is found to be 5.7×10^{17} cm^{-3}. Having obtained the 3D carrier density, we next compute the temperature dependent electron's mean free path following the well established approach described in the papers [6, 7].

In the Drude-Boltzmann picture, 3 dimensional conductivity (σ) of a metal with spherical Fermi surface is given by

$$\sigma = ne^2\tau/m^* = e^2(k_F)^2 l_e/3\pi^2\hbar \tag{4}$$

where n is the 3D carrier density given by n=$k_F{}^3/3\pi^2$ (k_F is the Fermi wavevector), and τ is scattering time constant given by τ=$l_e m^*/\hbar k_F$ where m^* is the effective mass of electrons and l_e is the electron's mean free path [8]. Rearranging the Supplementary Eq. (4), the expression for l_e can be written as

$$l_e = 3\pi^2\hbar/\rho(k_F)^2e^2 \tag{5}$$

where ρ=1/σ is the resistivity. Since all the measurements in the present study are performed in Van der Pauw geometry, the temperature dependent ρ is obtained from the formula

$$\rho = (\pi/ln2)Rt \tag{6}$$

where R is the measured temperature dependent resistance and t is the thickness of the conducting region. In the present case, t is calculated by equating the 3D carrier density obtained from the SdH analysis with the sheet carrier density n_S obtained from the Hall measurement divided by the thickness [n=n_S/t, see the reference [3]]. Apart from the ρ, k_F is the another input parameter in Supplementary Eq. (5) for calculating l_e. Since the Supplementary Eq. (5) holds only for the spherical Fermi surface, we make the following approximation. Since the Fermi surface of KTO is only moderately anisotropic at such dilute carrier density, for simplification, the value of k_F has been calculated by effectively mapping the ellipsoidal Fermi surface onto a spherical Fermi surface wherein we equate the total volume of the three ellipsoids with a single spherical Fermi surface of radius k_F. The value of k_F is then estimated from the relation k_F=(3$\pi^2 n)^{1/3}$ [8]. Once we obtain k_F, the temperature dependent ρ obtained from the Supplementary Eq. (6) along with k_F is plugged in the Supplementary Eq. (5) and temperature dependent electron's mean free path is calculated.

l_e calculated using this approach for the oxygen deficient KTO has been shown by a blue curve in the Fig. 1d of the main text. As evident from the plot, above $\sim$130 K, l_e becomes shorter than the inverse of k_F ($k_F l_e$ $>$ 1) and the system enters into a bad metal phase. A further correction to the calculated l_e is required above the Fermion degeneracy temperature above which the thermal de Broglie wavelength (given by Λ=h/$\sqrt{2\pi m^* k_B T}$) becomes larger than the inter electron separation given by $n^{-1/3}$. Above this temperature, electrons become non-degenerate and the modified l_e is given by $l'_e\sim$ l_e $n^{-1/3}$/Λ [6, 7]. For calculation of l'_e, one requires an estimation of m^*. In the present work we estimate the m^* by analyzing the temperature dependent SdH oscillations and is found to be 0.56 m_e (please refer to the section B of the supplemental material of our earlier paper [2] for more details). The orange curve in Fig. 1d of the main text shows the calculated l_e for the non-degenerate case. As evident, this exercise only shifts the curve to a little higher temperature and crossover to bad metal phase occurs around 145 K, however, the conclusions remain the same. In the current work, observation of glassy dynamics is inherently constrained to the temperatures which is much lower than the crossover temperature to bad metal phase and hence for all practical purposes our electron doped KTO system is in good metal regime with well defined scattering.

Supplementary Note 2: Sheet resistance vs temperature plot.

As depicted in Fig. 2b of the main text, shining light above 150 K does not have any noticeable effect. This observation is further supported by the plot shown in Supplementary Fig. 2, where we compare the R_S vs T curve obtained in the dark (taken in the cooling run) with the measurement conducted during the heating run after exposing the sample to green light (power= 145 μ Watt) for 30 minutes at 10 K. It is evident from the plot that the two curves converge around 150 K, indicating a lack of any significant photo-doping above 150 K.

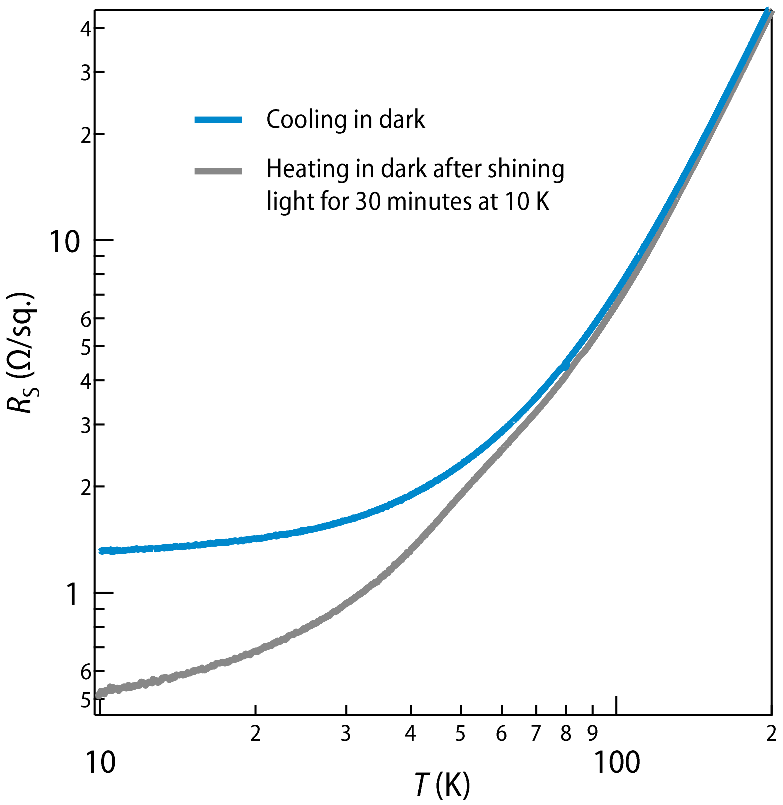

Supplementary Figure 2: Temperature dependent sheet resistance (R_S) of oxygen-deficient $KTaO_3$ sample (n=5.7×10^{17} cm^{-3}) taken in a cooling run in dark (sky blue curve). The slate-gray color curve shows data taken in a heating run after shining light for 30 minutes at 10 K.

Supplementary Note 3: Stretched exponential behavior of resistance relaxation in the light off stage.

The panels a-f in the Supplementary Fig. 3 show the temporal evolution of resistance before and after turning off the light along with the fitting in light off stage with an stretched exponential function (exp(-$(t/\tau)^{\beta}$) where τ is the relaxation time and β (stretching exponent) < 1). As evident, this function provides an excellent fit to experimental data at range of temperatures.

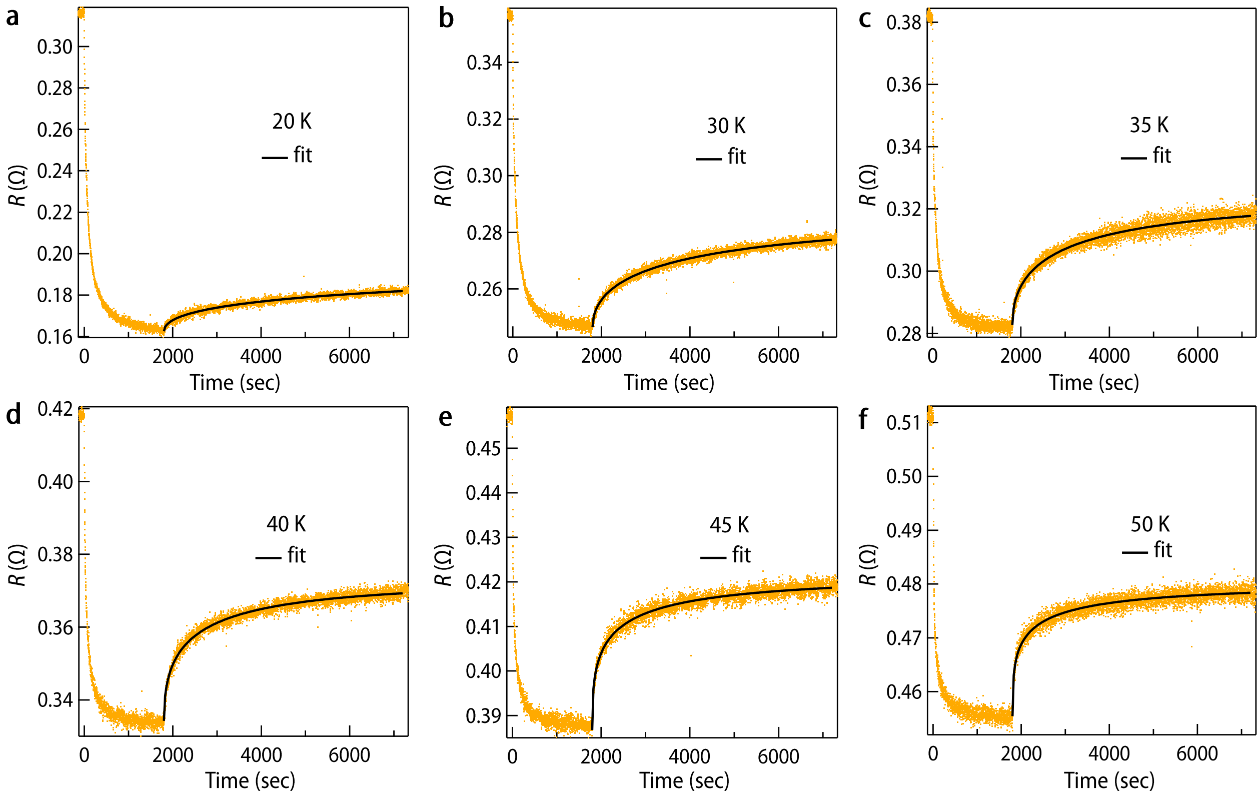

Supplementary Figure 3: Fitting of resistance relaxation in the light off stage with stretched exponential function at several fixed temperatures.

Supplementary Note 4: Power law behavior of persistence photo-resistance relaxation time (τ).

Supplementary Fig. 4 shows *log*-*log* plot of relaxation time (τ) as a function of temperature. As evident, the plot looks linear in wide range of temperatures belo 50 K signifying a power law dependence ($\tau \sim T^{-\alpha}$). The value of alpha obtained from fitting is found to be 2.8. We also emphasize that such power-law divergence of relaxation time has been also discussed theoretically in context of α relaxation in glasses [9].

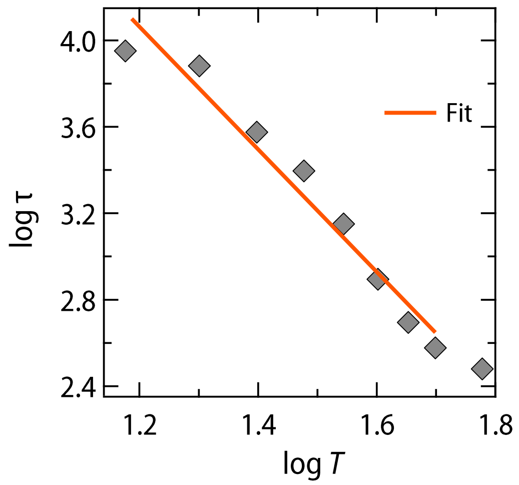

Supplementary Figure 4: Power law temperature dependence of relaxation time (τ).

Supplementary Note 5: Experiment with red light.

In the main text, we have presented all the measurements conducted using the green light. In this section, we provide an additional set of data obtained in three consecutive cycles (see Supplementary Fig. 5a) using a red light of wavelength λ = 650 nm, power = 60 μ Watt). Supplementary Fig. 5b and 5c shows the temperature evolution of β and τ respectively obtained from the fitting. As evident, while the enhancement in τ below 50 K is consistent with results with green light, β slightly decreases from its peak value at lower temperatures. However, this is within the error bar and the results qualitatively align with the findings from the measurements using green light and suggest that the behavior observed is not specific to a particular wavelength of light.

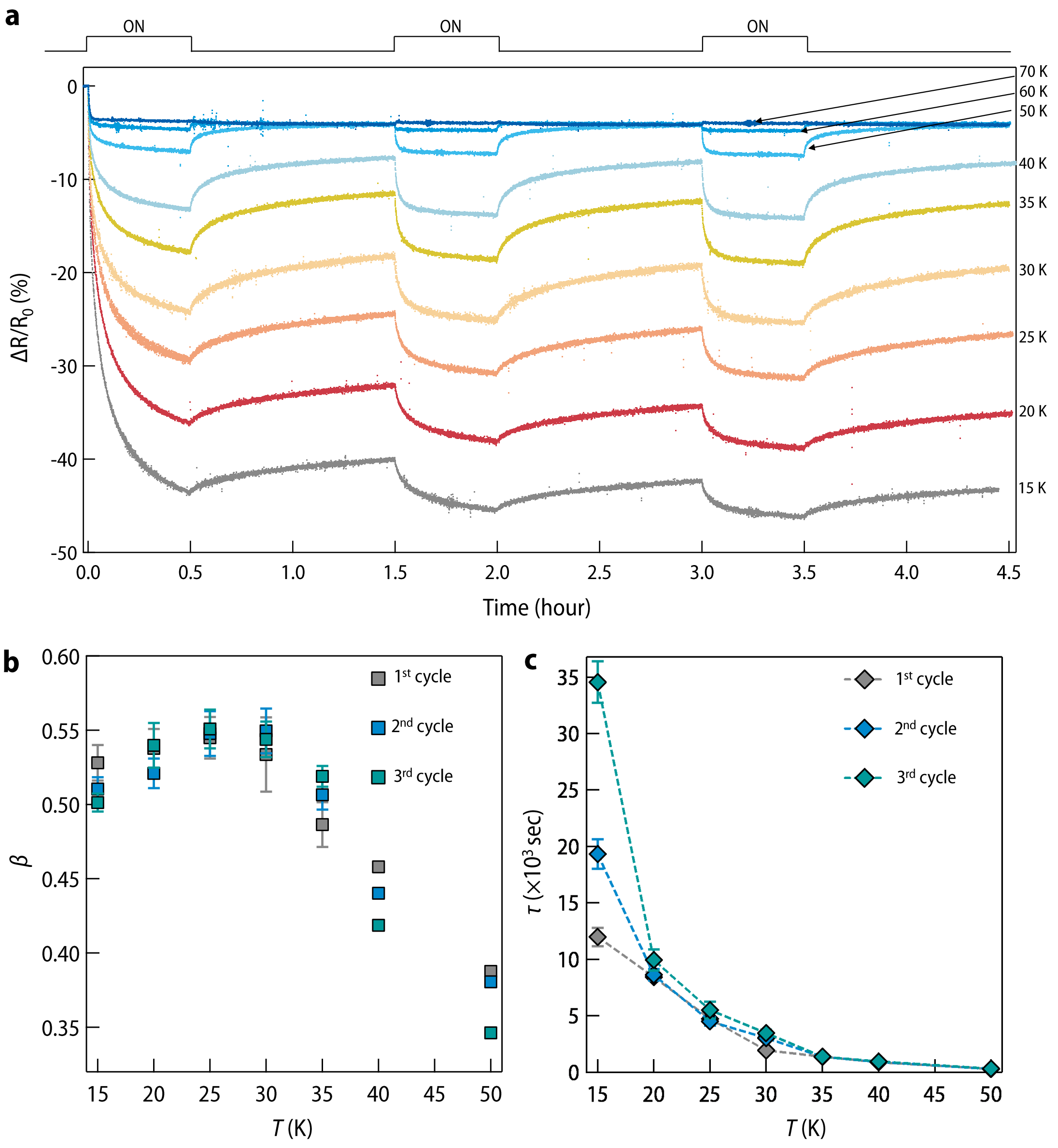

Supplementary Figure 5: **a.** Temporal evolution of resistance under red light illumination (λ = 650 nm, power = 60 μ watt) for 30 minutes measured at several fixed temperatures. After 30 minutes, resistance relaxation was observed in dark for the next one hour. This measurement was repeated for 3 consecutive cycles. For comparative analysis, change in resistance has been converted into relative percentage change $(\Delta R/R_0)\times 100$. **b.** Temperature dependence of stretching exponent (β) obtained from fitting of resistance relaxation in light off stage with a stretched exponential function for all three cycles. **c.** Temperature dependence of corresponding relaxation time (τ) for all three cycles.

Supplementary Note 6: Long-time relaxation of persistence photo-resistance.

In Fig. 2c of the main text and Supplementary Fig. 5b, the value of the stretching exponent was determined by fitting the resistance relaxation during the off-stage, which lasted for 1.5 hours. To ensure that the resistance relaxation was substantial enough to yield a reliable fitting, we conducted an additional measurement where the resistance relaxation was observed for an entire day (see Supplementary Fig. 6). It is important to emphasize that even when the data is fitted for an extended period of up to 24 hours, the same value of β = 0.5 is obtained.

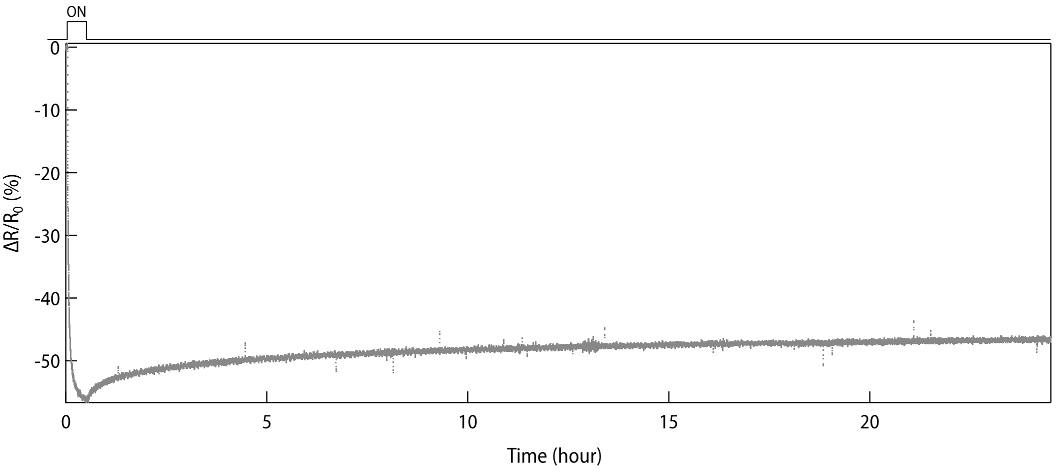

Supplementary Figure 6: Temporal evolution of resistance under green light illumination (λ = 527 nm, power = 145 μ Watt) for 30 minutes measured at 15 K. After 30 minutes, resistance relaxation was observed in dark for the next 24 hours. For comparative analysis, change in resistance has been converted into relative percentage change $(\Delta R/R_0)\times 100$.

Supplementary Note 7: Deviation of persistence photo-resistance relaxation time (τ) from activated behavior at low temperature.

In the large lattice relaxation (LLR) model [10], the recombination of electron-hole pairs occurs through the thermal excitation of electrons over an energy barrier. This process is an activated process of the Arrhenius type. To validate this model, we have plotted ln τ against 1000/T in Supplementary Fig. 7. It is evident from the plot that the data does not exhibit the expected linear behavior at low temperatures signifying that the LLR model can not account for our experimental observation of glassy dynamics below 35 K.

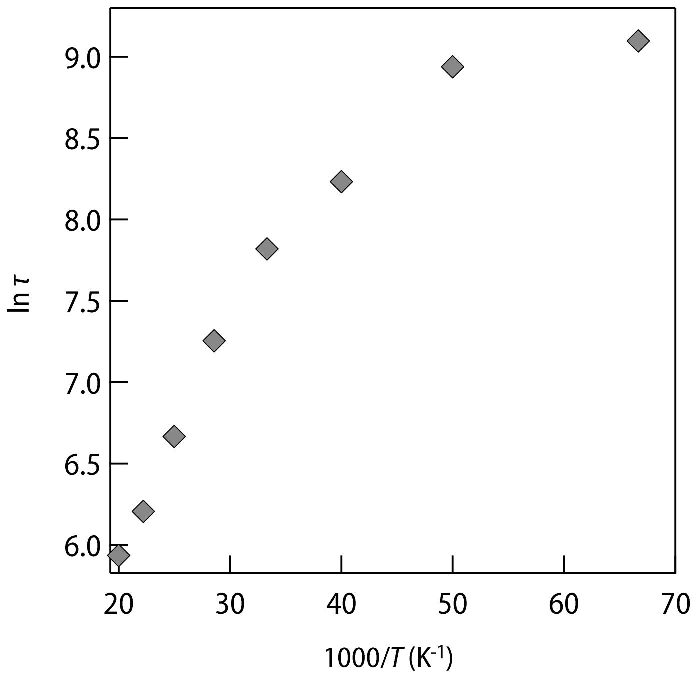

Supplementary Figure 7: Arrhenius plot of photo-resistance relaxation time (τ). As evident, a clear deviation from activation behavior is observed below 35 K.

Supplementary Note 8: Dielectric loss in pristine $KTaO_3$.

In its ideal form, $KTaO_3$ is perfectly centrosymmetric and hence, should not possess any electric dipole moment. However, the presence of impurities and disorder can disrupt the crystal's inversion symmetry, leading to the development of permanent electric dipoles [11, 12, 13, 14, 15, 16]. In highly polarizable host such as $KTaO_3$, these dipoles polarize the surrounding lattice leading to formation of polar nano regions (PNRs) [17]. Under an applied AC electric field, these PNRs act as a source of dielectric losses, which can be observed as a peak in the loss tangent ($\tan\delta = \epsilon''/\epsilon'$, where ϵ' and ϵ'' are the real and imaginary parts of the complex dielectric function, respectively).

Our temperature-dependent measurement of dielectric function indeed reveals the presence of PNRs even in our pristine $KTaO_3$ single crystal (see inset of Supplementary Fig. 8). Further, frequency-dependent measurements reveal that the dielectric loss is an activated process emphasizing that the PNRs in pristine crystals are very dilute and independent.

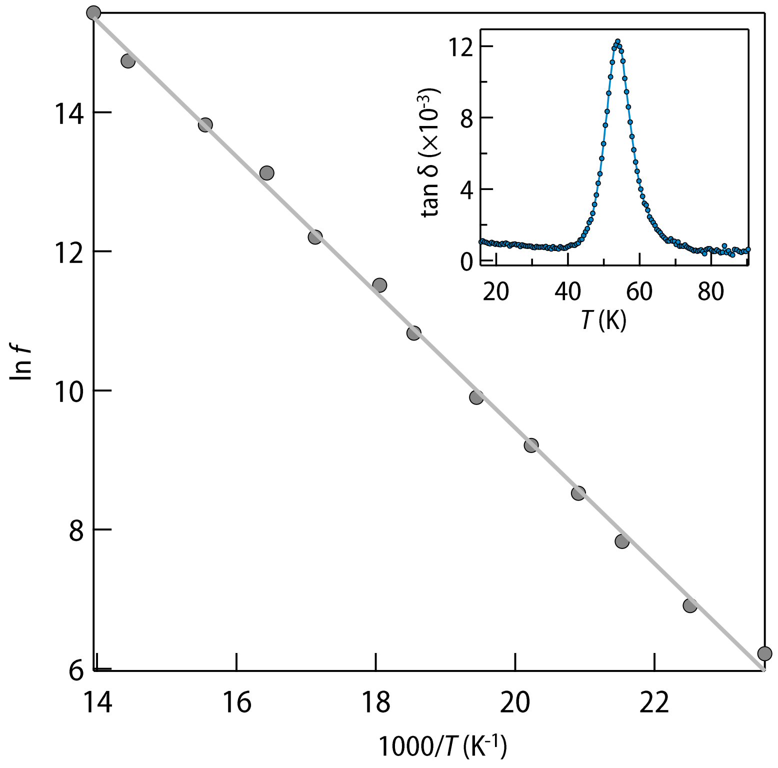

Supplementary Figure 8: Arrhenius plot of dielectric relaxation frequency (f) for pristine $KTaO_3$. Inset shows the dielectric loss tangent ($\tan\delta$) around 54 K at f = 50 kHz.

Supplementary Note 9. Correspondence between the appearance of polar nano regions and photo-doping effect.

In order to study the correlation between PNRs and photo-doping effect, we have compared the temperature-dependent total SHG intensity with the fraction of resistance (in terms of relative percentage change $(\Delta R/R_0)\times 100$) which has not been recovered at the end of 1.5 hours after turning off the light. We call this quantity the persistence photo resistance denoted with the symbol δ. See the inset of the bottom panel of Supplementary Fig. 9 for the definition of δ. As evident, the appearance of finite signal in SHG exactly coincides with the δ, strongly signifying the direct role of PNRs behind the effective electron-hole separation of our samples.

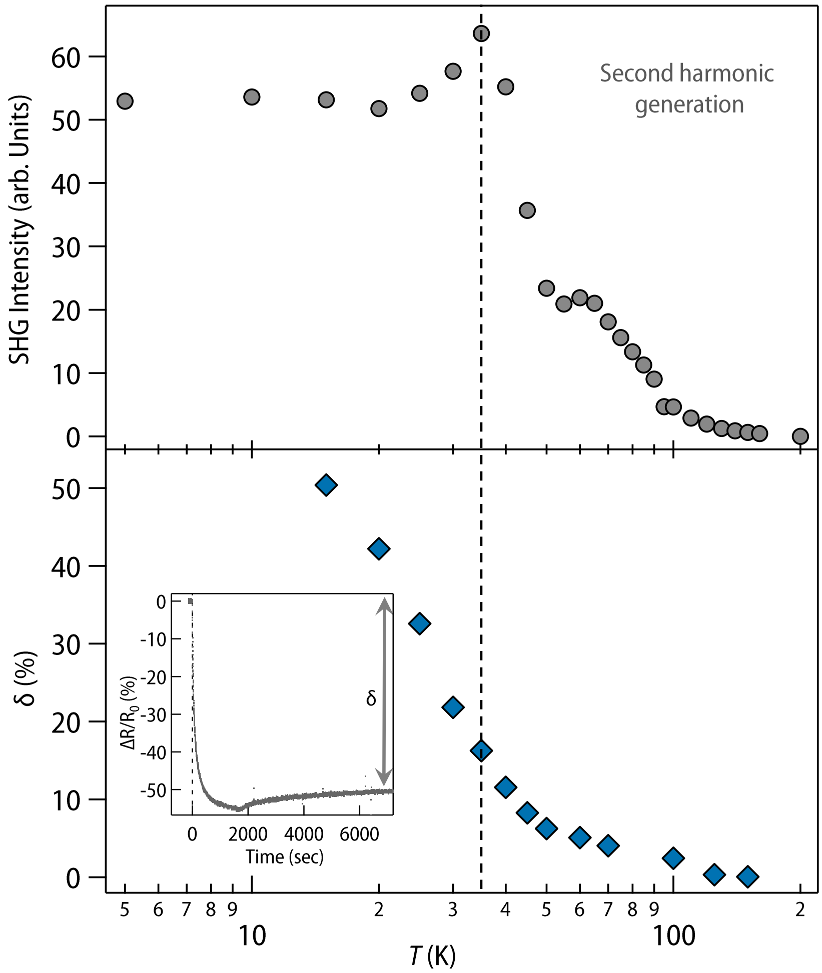

Supplementary Figure 9: (upper panel) Temperature-dependent SHG intensity measured on oxygen deficient $KTaO_3$ sample. (lower panel) Temperature dependence of persistence photo resistance (δ) in terms of relative percentage change at the end of 1.5 hours after turning off the light (see the inset of the lower panel for the definition of δ).

Supplementary Note 10: Raman measurement on a metallic oxygen deficient KTO.

Supplementary Fig. 10 shows the Raman spectra for one of the metallic oxygen-deficient KTO sample at room temperature. As evident, the soft polar mode (TO_1, marked by a red arrow) is preserved even in the metallic sample [18, 19, 20, 21, 22]. This result is consistent with our SHG measurement.

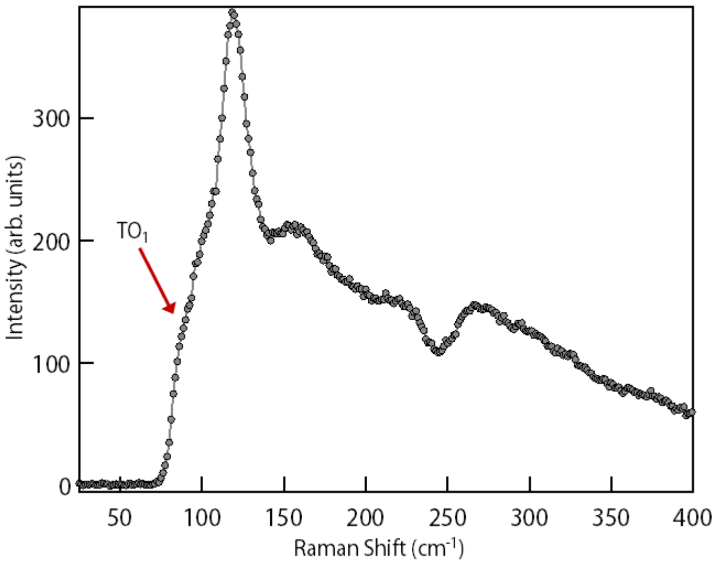

Supplementary Figure 10: Raman spectra of a metallic oxygen deficient KTO sample at room temperature.

Supplementary Note 11: Presence of potassium vacancy.

To investigate the presence of potassium vacancy in our oxygen-deficient $KTaO_3$ sample, K 2p core levels were collected at the Hard X-ray Photoelectron Spectroscopy (HAXPES) beamline (P22) at PETRA III, DESY. Supplementary Fig. 11a shows one representative experimental data (recorded with an incident photon energy of 5800 eV at room temperature) along with its fitting with the convolution of Lorentzian and Gaussian functions. It is evident from the figure that, in addition to the K 2p 3/2 peak at 292.65 eV arising from the lattice, there is an extra peak appearing at 293.1 eV. It has been previously shown that the presence of an additional peak at a higher binding energy is a characteristic feature of a potassium vacancy in the system [23]. In order to further understand the potassium vacancy profile in our sample, we have carried out measurements with varying photon energy from 3400 eV to 5800 eV (see Table I for the values of mean free path (MFP) and mean escape depth (MED)). As evident from Supplementary Fig. 11b, there are hardly any significant alterations in the line shape of the spectra as the photon energy was increased. This observation indicates that the potassium vacancy profile is homogeneous throughout the bulk of the sample.

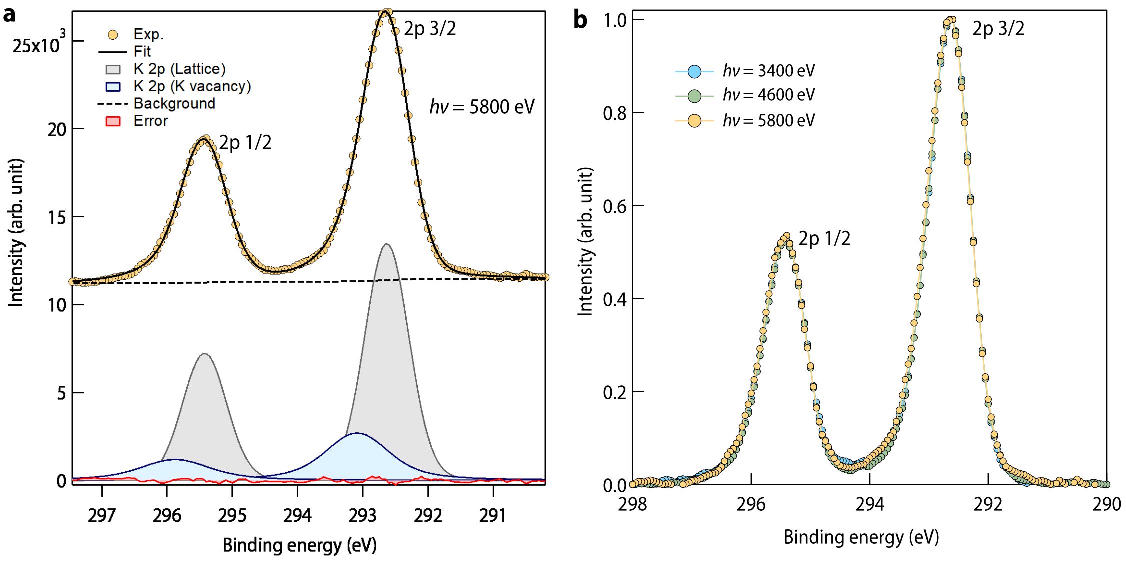

Supplementary Figure 11: **a** Deconvolution of K 2p core level spectrum (for oxygen-deficient $KTaO_3$ sample) by using the convolution of Lorentzian and Gaussian function. This data was recorded at room temperature with photon energy $h\nu$ = 5800 eV. Yellow-filled circles denote the experimental data and a solid black line denotes the simulated spectra. Curves filled with gray and sky blue color correspond to the lattice potassium and potassium vacancy respectively. The dashed black line denotes the Shirley background and the red-filled curve corresponds to the difference between the experimental data and simulated spectra. **b** K 2p core level spectra recorded with varying incident photon energy. All the spectra were recorded at room temperature.

Photon energy (eV)	MFP (nm)	MED (nm)
3400	5	15
4600	6.4	19.2
5800	7.7	23.1

Supplementary Table 1: Table of mean free path (MFP) and mean escape depth (MED) with increasing photo n energy. MFP wascalculated from the formula $m(\text{K.E.})^{\gamma}$ where K.E. is the kinetic energy of the ejected electrons and the values of$m$ and γ were taken to be 0.12 and 0.75 respectively [24]. Further, MED was roughly assumed to be 3 times MFPwhich would roughly account for 95 percent of the total collected intensity.

Supplementary Note 12: Berry phase calculations.

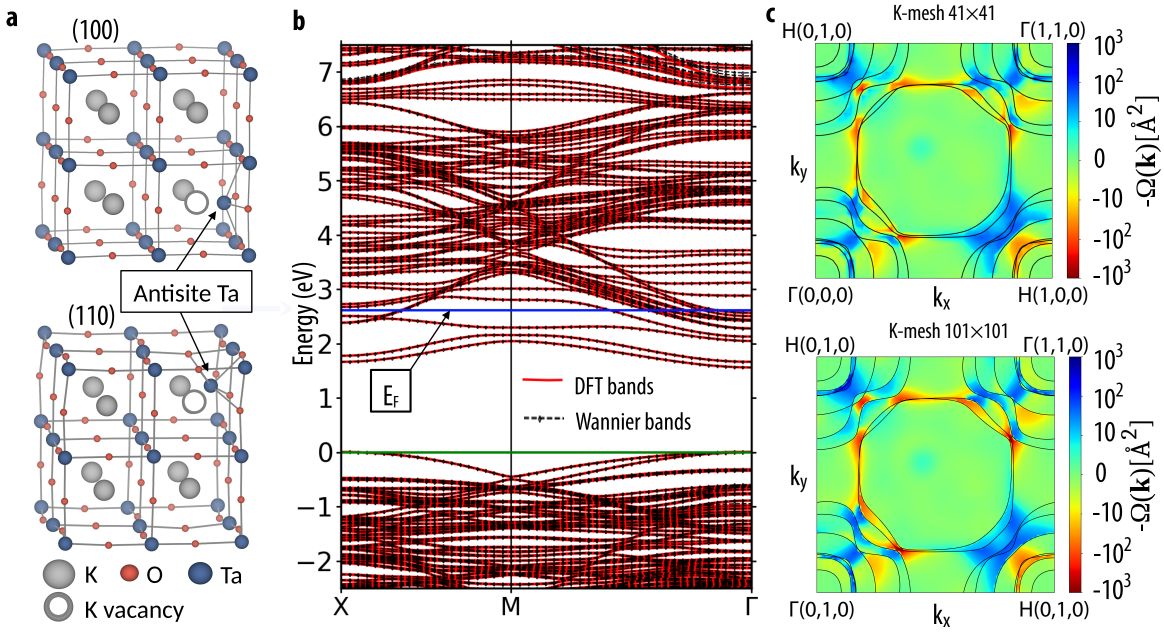

Supplementary Figure 12: **a.** The relaxed structure of $KTaO_3$ with Ta off-centering along the (100) and (110) direction around K vacancy (antisite like Ta defect) for a supercell of size 2×2×2. **b.** Band structure plot for (110) direction antisite like Ta defect calculated using density functional theory within PBE-GGA (shown in red solid lines) and Wannier interpolated bands (shown in black dotted lines). The blue solid line is the Fermi energy. **c.** Plot of total Berry curvature in the plane $k_z = 0$ (in log scale) for the (110) antisitelike Ta defect structure calculated on a 2D k-mesh of sample-size 41×41 (top panel) and 101×101 (bottom panel).

We have investigated the probable mechanism for the realization of PNRs theoretically using noncollinear density functional (DFT) theory. Antisite defect in perovskite materials like $SrTiO_3$ and complex perovskite oxides (Ca, $Sr)_3Mn_2O_7$ is known for causing macroscopic polarization in the system [25, 26]. Due to the similarities in the electronic properties of $SrTiO_3$ and $KTaO_3$, we expect $KTaO_3$ to develop polarization due to antsite defects. We have created Ta antisite defect in a supercell of size $2 \times 2 \times 2$ conventional unit cell. In our calculations, the Ta off-centering is considered along (100) and (110) directions. The relaxed structures are shown in Supplementary Fig. 12a. In Supplementary Fig. 12b we have shown the band structure of the system with Ta antisite defect along (110). The red solid lines are the bands calculated using DFT within PBE-GGA[27]. In order to calculate the macroscopic polarization in the system, we use the modern theory of polarization. The change in electronic contribution to the polarization ΔP is defined as [28, 29]

$$\Delta P = -\frac{e}{2\pi}\phi \tag{7}$$

where ϕ is the Berry phase, which is the integral of the Berry curvature over a surface S bounded by a closed path in $k-$space, *i.e.,* [30]

$$\phi = \int_S \Omega(\mathbf{k}) d\mathbf{k} \tag{8}$$

One can calculate Berry curvature using Bloch states $u_n(\mathbf{k})$ as

$$\Omega_{\alpha\beta}(\mathbf{k}) = \sum_n f_n(\mathbf{k})\Omega_{n,\alpha\beta}(\mathbf{k}) = \sum_n -2f_n(\mathbf{k})\mathrm{Im}\left\langle \frac{\partial u_n(\mathbf{k})}{\partial k_\alpha} \middle| \frac{\partial u_n(\mathbf{k})}{\partial k_\beta} \right\rangle \tag{9}$$

where α, β are the cartesian indices and $f_n(\mathbf{k})$ is the occupation number of the state n. Ta antisite defects in $KTaO_3$ have a partially occupied band. As it well known, taking derivatives of $u_n(\mathbf{k})$ in a finite-difference scheme in the presence of band crossing and avoided crossing becomes difficult. We followed the procedure described by X. Wang *et. al.* [31] for the calculation of Berry curvature using Wannier functions as implemented in the Wannier90 code [32]. In Supplementary Fig. 12b, we have shown the Wannier interpolated bands in black dotted lines. From the figure, it is clear that both the set of bands calculated using DFT and Wannier interpolation match

very well near the Fermi level. In Supplementary Fig. 12c, we have shown the total Berry curvature for the antisite Ta defect along (110) in the plane $k_z = 0$ calculated using 2D k-mesh of size 41×41 (top panel) and 101×101 (bottom panel). While there are small quantitative differences, the qualitative features in both the panels remain the same.

Supplementary Note 13: Toy model of complex inter-band electronic relaxation mediated by a glassy bath.

As discussed in the main text, we consider the following model of coupled electron (el)-glass (gl) system.

$$H = H_{el} + H_{gl} + H_{el-gl} \tag{10a}$$

$$H_{el} = -\sum_{i,j=1}^{N_c} t_{ij} c_i^\dagger c_j - \varepsilon_0 \sum_{\alpha=1}^{N_f} f_\alpha^\dagger f_\alpha, \quad (\varepsilon_0 > 0) \tag{10b}$$

$$H_{gl} = \sum_{\mu=1}^{N_g} \frac{p_\mu^2}{2m} + U(\{x_\mu\}) \tag{10c}$$

$$H_{el-gl} = \sum_{i\alpha\mu} (V_{i\alpha\mu} c_i^\dagger f_\alpha + \text{h.c.}) x_\mu \tag{10d}$$

The electronic part consists of a conduction band and a flat impurity band at energy $-\varepsilon_0$. We consider three different energy dispersions for the conduction band, corresponding to different lattices and hopping amplitudes t_{ij}, namely – (1) a flat band or δ function density of states (DOS) with bandwidth $W = 0$, (2) a semicircular DOS, $g(\epsilon) = (1/2\pi)\sqrt{W^2 - \omega^2}\theta(W - |\omega|)$ with band width W [$\theta(x)$ is heaviside step function], e.g., corresponding to a Bethe lattice, and (3) DOS corresponding to three-dimensional (3d) simple cubic lattice with nearest-neighbour hopping $W/12$. The band gap between the conduction band minimum and the impurity band is $\Delta = \varepsilon_0 - W/2$. We set the chemical potential at the center of the conduction band, i.e., $\mu = 0$, as appropriate for a metallic system (see Fig. 5(a) of main text.

The Hamiltonian H_{gl} for a set of particles with positions $\{x_\mu\}$, and their canonically conjugate momenta p_μ ($[x_\mu, p_\nu] = \imath\delta_{\mu\nu}$ with $\hbar = 1$) models the dynamics of a local glassy background. The exact form of the inter-particle interaction $U(\{x_\mu\})$ is not crucial for our calculations. However, as an example, we take $U(\{x_\mu\}) = \sum_{\mu<\nu<\gamma} J_{\mu\nu\gamma} x_\mu x_\nu x_\gamma$ with the spherical constraint $\sum_\mu x_\mu^2 = N_g$, corresponding a well-known solvable model for glasses, namely the infinite-range spherical p-spin glass model with $p = 3$-spin coupling [33]. Here, $J_{\mu\nu\gamma}$ is real Gaussian random number with zero mean and variance $\overline{J_{\mu\nu\gamma}^2} = 3!J^2/2N_g^2$, where the overline denotes averaging over different realizations of $J_{\mu\nu\gamma}$. The particular scaling with N_g ensures extensive free energy in the thermodynamic limit $N_g \to \infty$. The p-spin glass model, with both quantum [33] and classical dissipative [34] dynamics, undergoes a glass transition at temperature $T_g \sim J$. For temperature $T \gtrsim T_g$, the model gives rise to a supercooled liquid regime, like standard structural glasses [35], with complex two-step relaxation for the dynamical correlation function

$$C_{gl}(t) = \overline{\langle x_\mu(t) x_\mu(0)\rangle} = Ae^{-|t|/\tau_s} + Be^{-(|t|/\tau_\alpha)^\beta} \tag{11}$$

, having a short-time exponential decay, and long-time stretched exponential decay.

To obtain a solvable model for the coupled el-gl system, we also take the coupling $V_{i\alpha\mu}$ between the glass, and the conduction and impurity electronic states, infinite range. Here $V_{i\alpha\mu}$ is a complex Gaussian random number with zero mean and variance $\overline{|V_{i\alpha\mu}|^2} = V/(N_c N_f N_g)^{1/3}$. The particular scaling ensures a well-defined thermodynamic limit $N_c, N_f, N_g \to \infty$ with finite ratios $p_f = N_f/N_c$ and $p_g = N_g/N_c$. The ratios allow us tune the backaction of one part of the system on the other. For example, for simplicity, and to gain an analytical understanding, as discussed below, we take $p_f, p_g \gg 1$, such that the back actions of the conduction electrons on the impurity electrons and the glass are negligible. We expect our main conclusions to be valid for $p_f, p_g \approx 1$, i.e., when the backaction is substantial.

For the above-disordered model (Supplementary Eq. (10)), using the standard replica method [36, 33], we calculate the disorder-averaged connected equilibrium dynamical density-density correlation function for the conduction electrons, $C_{el}(t) = \overline{\langle n_i(t) n_i(0)\rangle} - \langle n_i(0)\rangle^2$, at temperature $T \ll \Delta$. Here $n_i = c_i^\dagger c_i$ is the conduction electron density. The correlation function $C_{el}(t)$ captures the relaxation of the thermally excited electrons in the conduction band at a finite temperature.

We obtain the replicated partition function, $Z^n = \int \mathcal{D}(\bar{c}, c)\mathcal{D}(\bar{f}, f)\mathcal{D}x \exp\big(-S[\bar{c}, c, \bar{f}, f, x]\big)$, as coherent-state imaginary-time path integral over the fermionic Grassmann fields $\{\bar{c}_{ia}, c_{ia}, \bar{f}_{\alpha a}, f_{\alpha a}\}$ and position variables

$\{x_{ia}\}$, with replica index $a = 1, \cdots, n$ and the action,

$$\begin{aligned} S_n = &\int_0^{1/T} d\tau \sum_{ij,a} \bar{c}_{ia}(\tau)[(\partial_\tau - \mu)\delta_{ij} - t_{ij}]c_{ja}(\tau) + \int_0^{1/T} d\tau \sum_{\alpha,a} \bar{f}_{\alpha a}(\tau)(\partial_\tau - \mu - \varepsilon_0)f_{\alpha a}(\tau) \\ &+ \int_0^{1/T} d\tau \left[\frac{1}{2}\sum_{\mu,a}[m(\partial_\tau x_{\mu a})^2 + z x_{\mu a}^2] + \sum_{\mu\nu\gamma,a} J_{\mu\nu\gamma} x_{\mu a}(\tau)x_{\nu a}(\tau)x_{\gamma a}(\tau)\right] \\ &+ \int_0^{1/T} d\tau \sum_{i\alpha\mu,a} [V_{i\alpha\mu}\bar{c}_{ia}(\tau)f_{\alpha a}(\tau)x_{\mu a}(\tau) + \text{h.c.}], \end{aligned} \tag{12}$$

where the Lagrange's multiplier $z(\tau)$ imposes the spherical constraint on $x_{\mu a}(\tau)$. After averaging over the distributions of $J_{\mu\nu\gamma}$ and $V_{i\alpha\mu}$, we obtain $\overline{Z^n}$ and the corresponding action as

$$\begin{aligned} \tilde{S}_n = &\int_0^{1/T} d\tau \sum_{ij,a} \bar{c}_{ia}(\tau)[(\partial_\tau - \mu)\delta_{ij} - t_{ij}]c_{ja}(\tau) + \int_0^{1/T} d\tau \sum_{\alpha,a} \bar{f}_{\alpha a}(\tau)(\partial_\tau - \mu - \varepsilon_0)f_{\alpha a}(\tau) \\ &+ \frac{1}{2}\int_0^{1/T} d\tau \sum_{\mu a} x_{\mu a}(\tau)(-m\partial_\tau^2 + z)x_{\mu a}(\tau) + N_c \int d\tau d\tau' \sum_{ab} \Big[V^2(p_f p_g)^{1/3} G_{ab}(\tau,\tau')\mathcal{G}_{ba}(\tau',\tau)Q_{ab}(\tau,\tau') \\ &- \frac{J^2 p_g}{4} Q_{ab}^3(\tau,\tau')\Big], \end{aligned} \tag{13}$$

where we have introduced the large N-fields,

$$G_{ab}(\tau,\tau') = -\frac{1}{N_c}\sum_i c_{ia}(\tau)\bar{c}_{ib}(\tau') \tag{14a}$$

$$\mathcal{G}_{ab}(\tau,\tau') = -\frac{1}{N_f}\sum_\alpha f_{\alpha a}(\tau)\bar{f}_{\alpha b}(\tau') \tag{14b}$$

$$Q_{ab}(\tau,\tau') = \frac{1}{N_g}\sum_\mu x_{\mu a}(\tau)x_{\mu b}(\tau') \tag{14c}$$

To promote the above as fluctuating dynamical fields, conjugate fields $\Sigma_{ba}(\tau',\tau)$, $\sigma_{ba}(\tau',\tau)$ and $\Pi_{ab}(\tau,\tau')$ are introduced for G, $\mathcal{G}$ and Q, respectively, e.g., by using the relation

$$\int \mathcal{D}G \prod_{a\tau,b\tau'} \delta(N_c G_{ab}(\tau,\tau') + \sum_i c_{ia}(\tau)\bar{c}_{ib}(\tau')) = \int \mathcal{D}G\mathcal{D}\Sigma e^{-\int d\tau d\tau' \Sigma_{ba}(\tau',\tau)[N_c G_{ab}(\tau,\tau') + \sum_i c_{ia}(\tau)\bar{c}_{ib}(\tau')]} = 1 \tag{15}$$

As a result, we can now integrate out the fields $(\bar{c}, c)$, $(\bar{f}, f)$, and x. Assuming replica diagonal ansatz, e.g., $G_{ab}(\tau,\tau') = \delta_{ab}G(\tau,\tau')$, we obtain $\overline{Z^n} = \int \mathcal{D}(G,\mathcal{G},Q,\Sigma,\sigma,\Pi)e^{-nS_{eff}}$ and the effective action

$$\begin{aligned} S_{\text{eff}} = &-N_c \int d\epsilon g(\epsilon)\,\text{Tr}\ln\big(-\partial_\tau + \mu - \epsilon - \Sigma\big) - N_f\,\text{Tr}\ln\big(-\partial_\tau + \mu + \varepsilon_0 - \sigma\big) + \frac{N_g}{2}\,\text{Tr}\ln\big(-m\partial_\tau^2 + z - \Pi\big) \\ &- N_c \int d\tau d\tau' \big[\Sigma(\tau,\tau')G(\tau',\tau) + p_f\sigma(\tau,\tau')\mathcal{G}(\tau',\tau) - \frac{p_g}{2}\Pi(\tau,\tau')Q(\tau',\tau) + \frac{p_g J^2}{4}Q(\tau,\tau')^3 \\ &- V^2(p_f p_g)^{1/3}G(\tau,\tau')\mathcal{G}(\tau',\tau)Q(\tau,\tau')\big] \end{aligned} \tag{16}$$

In the large N_c limit, the saddle point solution is obtained by varying the above action with respect to $G, \Sigma, \mathcal{G}, \sigma, Q, \Pi$ and setting the variations to zero. Due to time-translation invariance at equilibrium, e.g., $G(\tau,\tau') = G(\tau-\tau')$ and $z(\tau) = z$. As a result, we can write the saddle point equations in the following form after performing a Matsubara Fourier transform first, e.g., $G(\tau - \tau') \to G(\imath\omega_n)$ with fermionic Matsubara frequency ω_n, and then doing an analytical continuation, $G(\imath\omega_n) \to G_R(\omega + \imath 0^+)$, to real frequencies, where G_R is the retarded Green's function.

$$G_R(\omega) = \int d\epsilon g(\epsilon) \frac{1}{\omega + \mu - \epsilon - \Sigma_R(\omega)} \tag{17a}$$

$$\mathcal{G}_R^{-1}(\omega) = \omega + \mu + \varepsilon_0 - \sigma_R(\omega) \tag{17b}$$

$$Q_R^{-1}(\omega) = -m\omega^2 + z - \Pi_R(\omega) \tag{17c}$$

$$\Sigma(\tau) = V^2 (p_f p_g)^{1/3} \mathcal{G}(\tau) Q(\tau) \tag{17d}$$

$$\sigma(\tau) = V^2 p_g^{1/3} p_f^{-2/3} G(\tau) Q(\tau) \tag{17e}$$

$$\Pi(\tau) = \frac{3J^2}{2} Q^3(\tau) + V^2 p_f^{1/3} p_g^{-2/3} G(\tau) \mathcal{G}(-\tau) \tag{17f}$$

In the limit $p_f, p_g \gg 1$, we can approximate $\sigma(\tau) \approx 0$ and $\Pi(\tau) \approx (3J^2/2)Q^3(\tau)$, and neglect the back action of the conduction electrons on the impurity states and the glass.

In this limit, following the numerical procedure similar to that in reference [37, 38], we can numerically solve the above self-consistency equations for various conduction electron DOS $g(\epsilon)$ to obtain the retarded functions $G_R(\omega)$ and $Q_R(\omega)$, whereas the retarded Green's function of the impurity band is given by $\mathcal{G}_R(\omega) = (\omega + \imath 0^+ + \mu + \varepsilon_0)^{-1}$. However, instead of self-consistently solving for $Q_R(\omega)$, we use the Supplementary Eq. (11) to obtain the spectral function of the glass from the fluctuation-dissipation relation

$$\rho_{gl}(\omega) = -\frac{1}{\pi} \text{Im} Q_R(\omega) = -\frac{1}{\pi} \tanh\left(\frac{\omega}{2T}\right) C_{gl}(\omega), \tag{18}$$

where $C_{gl}(\omega) = \int_{-\infty}^{\infty} dt e^{\imath\omega t} C_{gl}(t)$. As a result, the conduction electron self-energy can be obtained as

$$\Sigma_R(\omega) = V^2 \int d\omega_1 d\omega_2 \rho_f(\omega_1) \rho_{gl}(\omega_2) \frac{n_F(\omega_1) n_B(\omega_2) - n_F(-\omega_1) n_B(-\omega_2)}{\omega - \omega_1 - \omega_2 + \imath 0^+}, \tag{19}$$

where $n_F(\omega)$ and $n_B(\omega)$ are Fermi and Bose functions, respectively, and $\rho_f(\omega) = \delta(\omega + \varepsilon_0)$ is the spectral function of the impurity electrons. Here we have redefined $V^2(p_f p_g)^{1/3}$ as V^2. Thus, using $\Sigma_R(\omega)$ in Supplementary Eq. (17a)), we can obtain the conduction electron Green's function $G_R(\omega)$.

0.1 Density density correlation function

To capture the manifestation of the glassy relaxation in the electron-hole recombination process we look into the connected density-density correlator $C_{el}(t) = \langle n_i(t) n_i(0) \rangle - \langle n_i(0) \rangle^2$, which captures the relaxation of the thermally excited carriers at temperature T. $C_{el}(t)$ can be obtained from the imaginary-time correlation function $C_{el}(\tau) = \langle n(\tau) n(0) \rangle - \langle n(0) \rangle^2$, where $n(\tau) = (1/N_c) \sum_i c_i^\dagger(\tau) c(\tau)$. In the large-$N_c$ limit, the connected correlator is given by the bubble diagram and can be expressed as $C_{el}(\tau) = G(\tau) G(-\tau)$. Performing Matsubara Fourier transformation and then analytically continuing $i\Omega_n \to \omega + i0^+$, where Ω_m is bosonic Matsubara frequency, we can obtain the retarded correlator

$$C_{el,R}(\omega) = \int d\omega_1 d\omega_2 \rho_c(\omega_1) \rho_c(\omega_2) \left(\frac{n_F(\omega_1) n_F(-\omega_2) - n_F(-\omega_1) n_F(\omega_2)}{\omega_1 - \omega_2 - \omega_n - i0^+} \right), \tag{20}$$

where $\rho_c(\omega) = -(1/\pi) \text{Im} G_R(\omega)$ is the conduction electron spectral function. Using the fluctuation-dissipation theorem, we can relate $C_{el,R}(\omega)$ to the Fourier transform of the real-time density-density correlation function $C_{el}(t)$ as $C_{el}(\omega) = \coth(\omega/2T) \text{Im} C_{el,R}(\omega)$. Finally, performing the inverse Fourier transform we obtain the real-time density-density correlation function $C_{el}(t) = \int_{-\infty}^{\infty} (d\omega/2\pi) e^{-i\omega t} C_{el}(\omega)$. We show the results for the numerically computed $C_{el}(t)$ in Fig. 5(c) of the main text for (1) a flat conduction band ($W = 0$), and (2) a semicircular conduction band DOS, using $C_{gl}(t)$ from Supplementary Eq. (11) (Fig. 5(b), main text). For the latter, we take a temperature-independent stretching exponent $\beta = 0.5$ and the α-relaxation time $\tau_\alpha(T)$, which varies as $\sim T^{-2.8}$, consistent with our experimental results (Fig. 2(c), main text). We also vary the coefficient $B(T) \leq 1$ such that it increases with decreasing temperature, while $A(T) = 1 - B(T)$. This leads to a more dominant long-time stretched exponential part in $C_{gl}(t)$ at lower temperatures, compared to the short-time exponential decay in Supplementary Eq. (11). In Supplementary Fig. 13, we show the results for the conduction band energy dispersion corresponding to a nearest-neighbour tight binding model on a simple cubic lattice. We can see that in all the cases the glassy relaxation of the bath influences the relaxation of the conduction electrons leading complex and temperature dependent relaxation profile for $C_{el}(t)$.

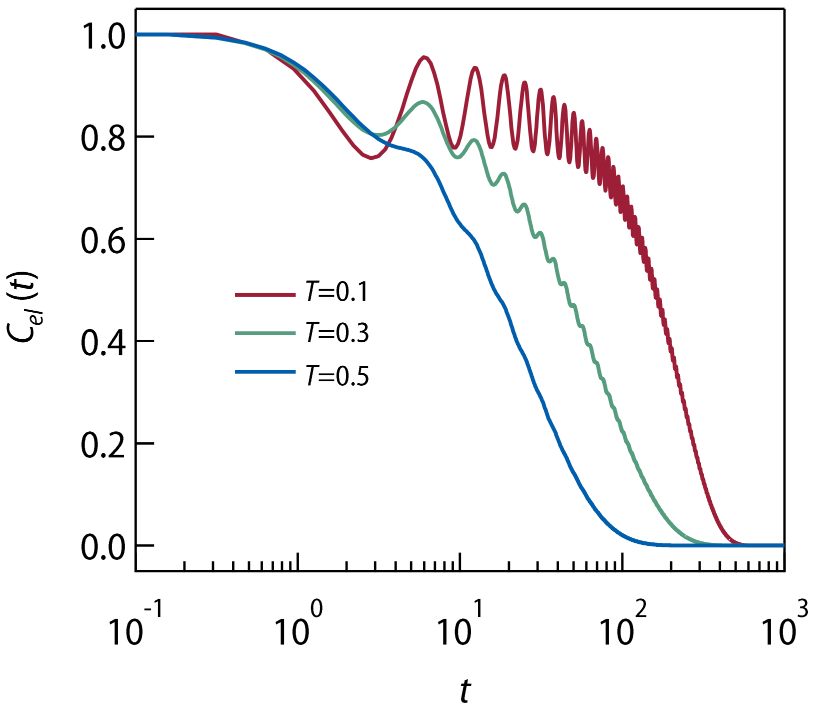

Supplementary Figure 13: The density-density correlation function of conduction electron $C_{el}(t)$ vs. t for three temperatures corresponding to a nearest-neighbour tight binding model on a simple cubic lattice

We can obtain a simple analytical understanding of the above results as follows. From Supplementary Eq. (19), we can obtain for $\mu = 0$

$$\gamma(\omega) = \mathrm{Im}\Sigma_R(\omega) = -\pi V^2 \rho_{gl}(\omega + \varepsilon_0)\big(n_F(-\varepsilon_0) + n_B(-\varepsilon_0 - \omega)\big) \tag{21}$$

Thus, by defining $k(\omega) = \mathrm{Re}\Sigma_R(\omega)$, we obtain from Supplementary Eq. (17a)

$$\mathrm{Im}G_R(\omega) = \int d\epsilon g(\epsilon) \frac{\gamma(\omega)}{(\omega - \epsilon - k(\omega))^2 + \gamma(\omega)^2} \tag{22}$$

As a result, from Supplementary Eq. (20) we get

$$\mathrm{Im}C_{el,R}(\omega) = \int d\omega_1 \rho_c(\omega_1)\rho_c(\omega_1 - \omega)\big(n_F(\omega_1) - n_F(\omega_1 - \omega)\big) \tag{23}$$

The above can be expressed as

$$\begin{aligned}\mathrm{Im}C_R(\omega) = \frac{1}{\pi^2}\int d\epsilon_1 d\epsilon_2 g(\epsilon_1)g(\epsilon_2)\int d\omega_1 \Bigg[&\frac{\gamma(\omega_1)}{(\omega_1 - \epsilon_1 - k(\omega_1))^2 + \gamma(\omega_1)^2} \\ &\frac{\gamma(\omega_1 - \omega)}{(\omega_1 - \omega - \epsilon_2 - k(\omega_1 - \omega))^2 + \gamma(\omega_1 - \omega)^2}\Bigg]\big(n_F(\omega_1) - n_F(\omega_1 - \omega)\big)\end{aligned} \tag{24}$$

Thus,

$$C_{el}(t) = \int_{-\infty}^{\infty} d\omega_1 d\omega e^{-\imath\omega t}\rho_{gl}(\omega_1 + \varepsilon_0)\rho_{gl}(\omega_1 + \varepsilon_0 - \omega)F(\omega, \omega_1) \tag{25}$$

can be written as a convolution over the glass spectral function $\rho_{gl}(\omega)$, where

$$\begin{aligned}F(\omega, \omega_1) = \frac{V^4}{2\pi}\int d\epsilon_1 d\epsilon_2 &\frac{g(\epsilon_1)g(\epsilon_2)\coth(\omega/2T)\,[n_F(\omega_1) - n_F(\omega_1 - \omega)]}{[(\omega_1 - \epsilon_1 - k(\omega_1))^2 + \gamma(\omega_1)^2][(\omega_1 - \omega - \epsilon_2 - k(\omega_1 - \omega))^2 + \gamma(\omega_1 - \omega)^2]} \\ &\big(n_F(-\varepsilon_0) + n_B(-\varepsilon_0 - \omega_1)\big)\big(n_F(-\varepsilon_0) + n_B(-\varepsilon_0 - \omega_1 + \omega)\big)\end{aligned} \tag{26}$$

The spectral function of the glass contains the information about the multiple time scales and the non-trivial temperature dependence of the glassy relaxation. Thus if appropriate conditions on the electronic energy scales W and ε_0 (Δ) are made relative to the energy scales of the glass, e.g., τ_s^{-1} and τ_α^{-1}, then the complex relaxation of the conduction electrons can be obtained. We numerically find that these conditions are met if the glassy bath is broad, i.e., the bandwidth of the glass is comparable or larger than the electronic energy scales.

References

[1] Mattheiss, L. F. Energy bands for knif$_3$, srtio$_3$, kmoo$_3$, and ktao$_3$. *Phys. Rev. B* **6**, 4718–4740 (1972). URL https://link.aps.org/doi/10.1103/PhysRevB.6.4718.

[2] Ojha, S. K. *et al.* Oxygen vacancy-induced topological hall effect in a nonmagnetic band insulator. *Advanced Quantum Technologies* **3**, 2000021 (2020). URL https://doi.org/10.1002/qute.202000021.

[3] Herranz, G. *et al.* High mobility in laalo$_3$/srtio$_3$ heterostructures: Origin, dimensionality, and perspectives. *Phys. Rev. Lett.* **98**, 216803 (2007). URL https://link.aps.org/doi/10.1103/PhysRevLett.98.216803.

[4] Uwe, H., Kinoshita, J., Yoshihiro, K., Yamanouchi, C. & Sakudo, T. Evidence for light and heavy conduction electrons at the zone center in ktao$_3$. *Phys. Rev. B* **19**, 3041–3044 (1979). URL https://link.aps.org/doi/10.1103/PhysRevB.19.3041.

[5] Herranz, G. *et al.* Full oxide heterostructure combining a high-T_c diluted ferromagnet with a high-mobility conductor. *Phys. Rev. B* **73**, 064403 (2006). URL https://link.aps.org/doi/10.1103/PhysRevB.73.064403.

[6] Lin, X. *et al.* Metallicity without quasi-particles in room-temperature strontium titanate. *npj Quantum Materials* **2**, 41 (2017). URL https://doi.org/10.1038/s41535-017-0044-5.

[7] Collignon, C., Bourges, P., Fauqué, B. & Behnia, K. Heavy nondegenerate electrons in doped strontium titanate. *Phys. Rev. X* **10**, 031025 (2020). URL https://link.aps.org/doi/10.1103/PhysRevX.10.031025.

[8] Ashcroft, N. W. & Mermin, N. D. *Solid state physics* (Cengage Learning, 2022).

[9] Kob, W. Supercooled liquids, the glass transition, and computer simulations. lecture notes" slow relaxation and nonequilibrium dynamics in condensed matter. In *Les Houches Session*, vol. 77 (2002).

[10] Lang, D. V. & Logan, R. A. Large-lattice-relaxation model for persistent photoconductivity in compound semiconductors. *Phys. Rev. Lett.* **39**, 635–639 (1977). URL https://link.aps.org/doi/10.1103/PhysRevLett.39.635.

[11] Grenier, P., Bernier, G., Jandl, S., Salce, B. & Boatner, L. A. Fluorescence and ferroelectric microregions in ktao3. *Journal of Physics: Condensed Matter* **1**, 2515 (1989). URL https://dx.doi.org/10.1088/0953-8984/1/14/007.

[12] Geifman, I. N. & Golovina, I. S. Discussion about the nature of polarized microregions in ktao3. *Ferroelectrics* **199**, 115–120 (1997). URL https://doi.org/10.1080/00150199708213433.

[13] Voigt, P. & Kapphan, S. Experimental study of second harmonic generation by dipolar configurations in pure and li-doped ktao3 and its variation under electric field. *Journal of Physics and Chemistry of Solids* **55**, 853–869 (1994). URL https://www.sciencedirect.com/science/article/pii/0022369794900108.

[14] Grenier, P., Jandl, S., Blouin, M. & Boatner, L. A. Study of ferroelectric microdomains due to oxygen vacancies in ktao3. *Ferroelectrics* **137**, 105–111 (1992). URL https://doi.org/10.1080/00150199208015942.

[15] Trybuła, Z., Miga, S., Łoś, S., Trybuła, M. & Dec, J. Evidence of polar nanoregions in quantum paraelectric ktao3. *Solid State Communications* **209-210**, 23–26 (2015). URL https://www.sciencedirect.com/science/article/pii/S0038109815000782.

[16] Tkach, A., Zlotnik, S. & Vilarinho, P. M. Dielectric response of ktao3 single crystals weakly co-doped with li and mn (2021). URL https://doi.org/10.3390/cryst11101222.

[17] Samara, G. A. The relaxational properties of compositionally disordered abo¡sub¿3¡/sub¿perovskites. *Journal of Physics: Condensed Matter* **15**, R367–R411 (2003). URL https://doi.org/10.1088/0953-8984/15/9/202.

[18] Shirane, G., Nathans, R. & Minkiewicz, V. J. Temperature dependence of the soft ferroelectric mode in ktao_3. *Phys. Rev.* **157**, 396–399 (1967). URL https://link.aps.org/doi/10.1103/PhysRev.157.396.

[19] Nilsen, W. G. & Skinner, J. G. Raman spectrum of potassium tantalate. *The Journal of Chemical Physics* **47**, 1413–1418 (2004). URL https://doi.org/10.1063/1.1712096.

[20] Perry, C. H., Fertel, J. H. & McNelly, T. F. Temperature dependence of the raman spectrum of srtio3 and ktao3. *The Journal of Chemical Physics* **47**, 1619–1625 (2004). URL https://doi.org/10.1063/1.1712142.

[21] Uwe, H., Lyons, K. B., Carter, H. L. & Fleury, P. A. Ferroelectric microregions and raman scattering in ktao_3. *Phys. Rev. B* **33**, 6436–6440 (1986). URL https://link.aps.org/doi/10.1103/PhysRevB.33.6436.

[22] Golovina, I. S. *et al.* Defect driven ferroelectricity and magnetism in nanocrystalline ktao3. *Physica B: Condensed Matter* **407**, 614–623 (2012). URL https://www.sciencedirect.com/science/article/pii/S0921452611011707.

[23] Kubacki, J., Molak, A., Rogala, M., Rodenbücher, C. & Szot, K. Metal–insulator transition induced by non-stoichiometry of surface layer and molecular reactions on single crystal ktao3. *Surface Science* **606**, 1252–1262 (2012). URL https://www.sciencedirect.com/science/article/pii/S0039602812001252.

[24] Pal, B., Mukherjee, S. & Sarma, D. D. Probing complex heterostructures using hard x-ray photoelectron spectroscopy (haxpes). *Journal of Electron Spectroscopy and Related Phenomena* **200**, 332–339 (2015). URL https://www.sciencedirect.com/science/article/pii/S0368204815001334.

[25] Jang, H. W. *et al.* Ferroelectricity in strain-free srtio_3 thin films. *Phys. Rev. Lett.* **104**, 197601 (2010). URL https://link.aps.org/doi/10.1103/PhysRevLett.104.197601.

[26] Miao, L. *et al.* Double-bilayer polar nanoregions and mn antisites in (ca, sr)3mn2o7. *Nature Communications* **13**, 4927 (2022). URL https://doi.org/10.1038/s41467-022-32090-w.

[27] Perdew, J. P., Burke, K. & Ernzerhof, M. Generalized gradient approximation made simple. *Phys. Rev. Lett.* **77**, 3865–3868 (1996). URL https://link.aps.org/doi/10.1103/PhysRevLett.77.3865.

[28] King-Smith, R. D. & Vanderbilt, D. Theory of polarization of crystalline solids. *Phys. Rev. B* **47**, 1651–1654 (1993). URL https://link.aps.org/doi/10.1103/PhysRevB.47.1651.

[29] Resta, R. Macroscopic polarization in crystalline dielectrics: the geometric phase approach. *Rev. Mod. Phys.* **66**, 899–915 (1994). URL https://link.aps.org/doi/10.1103/RevModPhys.66.899.

[30] Berry, M. V. Quantal phase factors accompanying adiabatic changes. *Proceedings of the Royal Society of London. A. Mathematical and Physical Sciences* **392**, 45–57 (1984). URL https://royalsocietypublishing.org/doi/abs/10.1098/rspa.1984.0023. https://royalsocietypublishing.org/doi/pdf/10.1098/rspa.1984.0023.

[31] Wang, X., Yates, J. R., Souza, I. & Vanderbilt, D. Ab initio calculation of the anomalous hall conductivity by wannier interpolation. *Phys. Rev. B* **74**, 195118 (2006). URL https://link.aps.org/doi/10.1103/PhysRevB.74.195118.

[32] Pizzi, G. *et al.* Wannier90 as a community code: new features and applications. *Journal of Physics: Condensed Matter* **32**, 165902 (2020). URL https://dx.doi.org/10.1088/1361-648X/ab51ff.

[33] Cugliandolo, L. F., Grempel, D. R. & da Silva Santos, C. A. Imaginary-time replica formalism study of a quantum spherical p-spin-glass model. *Phys. Rev. B* **64**, 014403 (2001). URL https://link.aps.org/doi/10.1103/PhysRevB.64.014403.

[34] Crisanti, A. & Sommers, H. J. The sphericalp-spin interaction spin glass model: the statics. *Zeitschrift fur Physik B Condensed Matter* **87**, 341–354 (1992).

[35] Kob, W. The Mode-Coupling Theory of the Glass Transition. *arXiv e-prints* cond–mat/9702073 (1997). cond-mat/9702073.

[36] Marinari, E., Parisi, G. & Ritort, F. Replica field theory for deterministic models. ii. a non-random spin glass with glassy behaviour **27**, 7647 (1994). URL https://dx.doi.org/10.1088/0305-4470/27/23/011.

[37] Banerjee, S. & Altman, E. Solvable model for a dynamical quantum phase transition from fast to slow scrambling. *Phys. Rev. B* **95**, 134302 (2017). URL https://link.aps.org/doi/10.1103/PhysRevB.95.134302.

[38] Bera, S., Venkata Lokesh, K. Y. & Banerjee, S. Quantum-to-classical crossover in many-body chaos and scrambling from relaxation in a glass. *Phys. Rev. Lett.* **128**, 115302 (2022). URL https://link.aps.org/doi/10.1103/PhysRevLett.128.115302.
